# Polymorphisms in signal transducer and activator of transcription 3 and lung function in asthma

**DOI:** 10.1186/1465-9921-6-52

**Published:** 2005-06-03

**Authors:** Augusto A Litonjua, Kelan G Tantisira, Stephen Lake, Ross Lazarus, Brent G Richter, Stacey Gabriel, Eric S Silverman, Scott T Weiss

**Affiliations:** 1 Channing Laboratory, Department of Medicine, Brigham and Women's Hospital, 181 Longwood Avenue, Boston, MA 02115 USA; 2 Division of Pulmonary and Critical Care Medicine, Beth Israel Deaconess Medical Center, 330 Brookline Avenue, Boston, MA 02215 USA; 3 Pulmonary Division, Brigham and Women's Hospital, 75 Francis Street, Boston, MA 02115 USA; 4 Harvard Medical School, 25 Shattuck Street, Boston, MA 02115 USA; 5 Whitehead Institute, Massachusetts Institute of Technology, Nine Cambridge Center, Cambridge, MA 02142 USA

## Abstract

**Background:**

Identifying genetic determinants for lung function is important in providing insight into the pathophysiology of asthma. Signal transducer and activator of transcription 3 is a transcription factor latent in the cytoplasm; the gene (*STAT3*) is activated by a wide range of cytokines, and may play a role in lung development and asthma pathogenesis.

**Methods:**

We genotyped six single nucleotide polymorphisms (SNPs) in the *STAT3 *gene in a cohort of 401 Caucasian adult asthmatics. The associations between each SNP and forced expiratory volume in 1 second (FEV_1_), as a percent of predicted, at the baseline exam were tested using multiple linear regression models. Longitudinal analyses involving repeated measures of FEV_1 _were conducted with mixed linear models. Haplotype analyses were conducted using imputed haplotypes. We completed a second association study by genotyping the same six polymorphisms in a cohort of 652 Caucasian children with asthma.

**Results:**

We found that three polymorphisms were significantly associated with baseline FEV_1_: homozygotes for the minor alleles of each polymorphism had lower FEV_1 _than homozygotes for the major alleles. Moreover, these associations persisted when we performed an analysis on repeated measures of FEV_1 _over 8 weeks. A haplotypic analysis based on the six polymorphisms indicated that two haplotypes were associated with baseline FEV_1_. Among the childhood asthmatics, one polymorphism was associated with both baseline FEV_1 _and the repeated measures of FEV_1 _over 4 years.

**Conclusion:**

Our results indicate that genetic variants in *STAT3*, independent of asthma treatment, are determinants of FEV_1 _in both adults and children with asthma, and suggest that *STAT3 *may participate in inflammatory pathways that have an impact on level of lung function.

## Background

It is recognized that genetic factors influence lung function [1,2]. The identification of genetic variants that determine either lung function development or decline is particularly important for diseases in which low lung function is a feature, such as chronic obstructive pulmonary disease and asthma, since this provides insight into the pathophysiology of these disorders. This may also be relevant to non-pulmonary disorders that have been associated with low lung function, such as cardiovascular disorders [3,4] and diabetes [5], in which these genes may control systemic mechanisms (e.g. inflammation) that contribute to both low lung function and disease development.

Signal transducer and activator of transcription 3 (STAT3) is a member of a protein family of transcription factors, which was discovered in the course of studies of interferon-induced intracellular signal transduction [6]. These proteins are latent in the cytoplasm and become activated through tyrosine phosphorylation which typically occurs through cytokine receptor associated kinases (the Janus kinase-signal transducer or JAKs). The JAK-STAT pathway transmits information received from extracellular polypeptide signals, through membrane receptors, directly to target gene promoters in the nucleus, providing a mechanism for transcriptional regulation without second messengers. The gene, *STAT3*, is induced by a wide-array of cytokines, including interleukin (IL)-6, IL-10, and IL-13, and has been implicated in the regulation of cell growth, inflammation, immune tolerance and early embryonic development. Recently, *STAT3 *was implicated in asthma pathogenesis in a study that showed that STAT3-dependent pathways induced by IL-13 in lung myofibroblasts were inhibited by the administration of the inhaled corticosteroid, fluticasone [7]. This suggests a role in airway inflammation and remodeling in asthmatics, which may affect lung function level.

In a previous study of the pharmacogenetics of asthma treatment, *STAT3 *was one of the candidate genes that we screened for association with response to cortocosteroid treatment [8]. Single nucleotide polymorphisms (SNPs) in *STAT3 *were genotyped and tested in a screening dataset from an adult asthma clinical trial. No effect of *STAT3 *SNPs on asthma drug response was seen in that study. However, the polymorphisms affected baseline lung function. In this report, we present our analysis of the association of *STAT3 *SNPs with lung function in adults with asthma, and replicate our findings in a cohort of children with asthma.

## Methods

### Populations and Study Samples

We used information from two asthma clinical trials, as previously reported [8]. All patients or their legal guardians consented to the study protocol and ancillary genetic testing. The Adult Study was a multicenter 8-week randomized clinical trial comparing the effect of once-daily high-dose inhaled flunisolide therapy with that of standard inhaled corticosteroid therapy (i.e. high vs standard dose inhaled corticosteroid therapy) among moderate to severe adult asthmatics [8]. Inclusion criteria were a history of asthma, ≥ 12% improvement in FEV_1 _with albuterol, and use of inhaled steroids at randomization. Exclusion criteria were non-asthma pulmonary disease, smoking (≥ 10 pack-years), and recent asthma exacerbations requiring systemic steroids. Subjects were phoned weekly and had spirometry at 4 and 8 weeks. For this analysis, we included only the 401 Caucasian participants. The Childhood Asthma Management Program (CAMP) is a multicenter, randomized, double-blinded clinical trial testing the safety and efficacy of inhaled budesonide vs. nedocromil vs. placebo over a mean of 4.3 years. Trial design and methodology for CAMP have been published [9,10]. CAMP enrolled 1,041 children ages 5 to 12 years with mild to moderate asthma. Entry criteria included asthma symptoms and / or medication use for ≥ 6-months in the previous year and airway responsiveness with a provocative concentration of methacholine causing a 20% reduction in FEV_1 _(PC_20_) ≤ 12.5 mg/ml. Data for 652 Caucasian children were included in this analysis.

### Phenotypes

The primary phenotype of interest in both cohorts was baseline, pre-bronchodilator forced expiratory volume in one second, as a percentage of predicted (PPFEV_1_). In the Adult Study, baseline PPFEV_1 _was measured after an open-label 4-week period that demonstrated stability on the study drug or on standard inhaled therapy. Spirometry was then performed at monthly intervals, for a total of three spirometric measurements. In CAMP, baseline spirometry was performed at randomization, after a 28-day period during which only as-needed albuterol was allowed. Follow-up spirometry was perfomed at 2, 4, 12, 16, 24, 28, 36, 40, and 48 months. In addition to the analysis of the baseline PPFEV_1_, a repeated measures analysis was performed in both cohorts, making use of the longitudinal follow-up for each subject. In CAMP, we included information on parental smoking obtained from the baseline questionnaire.

### SNP Selection and Genotyping

**Figure 1:**
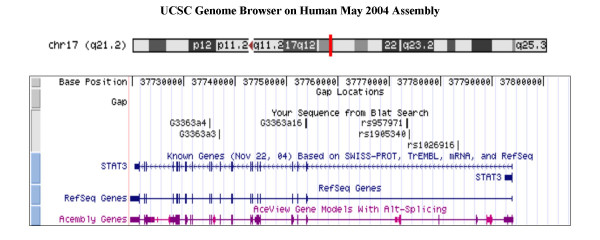
**Positions of the *STAT3 *SNPs genotyped in both the Adult Study and the CAMP cohort. **From UCSC Genome Browser , May 2004 Human Reference Sequence Assembly[11,12]. UCSC webpage was accessed on March 18, 2005.

SNPs were selected from two sources, public databases and genomic DNA sequencing performed at the Whitehead Institute. Three SNPs were discovered as a result of the sequencing effort: G3363a3, G3363a4, and G3363a16. These three SNPs have been submitted to the public database and correspond precisely to rs8075442, rs2293152, and rs2306581, respectively (dbSNP: ). Five additional SNPs were chosen from public databases for genotyping, with the overall goal of having, on average, at least one SNP every 10 kilobases. Two of these SNPs – rs1803125 (exonic) and rs744284 (promoter) – were found to be monomorphic in the Adult Study subjects, and were not subsequently genotyped in the CAMP cohort. Three additional SNPs were successfully genotyped in both cohorts: rs1026916, rs1905340, and rs957971. These are all intronic SNPs (Figure 1[11,12]), and flanking sequences are given in Table 1. Linkage disequilibrium (LD) between each pair of SNPs was calculated and plotted using the LDPlotter tool , and expressed as the r^2 ^LD statistic [13].

**Table 1:** Flanking sequences for *STAT3 *SNPs

STAT3	G3363a16	GGGAAAATGAGATCAGGAGATAAAG [G/T]GGCACCCTTTGGTCTTGTAAAGCCTTTTTTA
STAT3	G3363a3	ACAGACATCATTTGAACTAGAGACTCT [G/A]TCTTTATTCAGAGATCTTCATTTTGTGGAC
STAT3	G3363a4	TCCCCTTCACAAAGGGCCTCTGGCTGC [C/G]GGAGAGGGCTAGGGAGAGCCTCACAG
STAT3	rs1026916	AGGAAAAAGTTTAACCCAAAGACTGT [A/G]TGGATCTTCTCTACCCTACATCTCCAATCT
STAT3	rs1905340	TATTTGAGAATCTAAGAAAGTAGATCA [A/C]ACTAAATATTGATATGCAGACACTAAAATC
STAT3	rs957971	TGTTATATGAAGTGAATTAACCTCCTAT [C/G]GTACTTCAGTTTTCTCTATGCTAAAAGTGT

SNPs were genotyped via a SEQUENOM MassARRAY MALDI-TOF mass spectrometer (Sequenom, San Diego, CA) for analysis of unlabeled single-base extension minisequencing reactions with a semiautomated primer design program (SpectroDESIGNER, Sequenom). Our protocol implemented the very short extension method [14], whereby sequencing products are extended by only one base for three of the four nucleotides and by several additional bases for the fourth nucleotide (representing one of the alleles for a given SNP), permitting clearly delineated mass separation of the two allelic variants at a given locus.

### Statistical Analysis

Single SNP association analyses were performed with SAS statistical software (SAS Institute, Inc., Cary, NC). Univariate associations between SNPs and the phenotype of interest (PPFEV_1_) were tested by univariate linear regression, as implemented in Proc Reg in SAS. Multivariable linear regression models, were used to control for potential confounders. In these models, the genotype for each SNP was coded as three-level categorical variables (additive genetic model) or as dummy-coded variables. Hardy-Weinberg equilibrium for each SNP was tested using the chi-square goodness-of-fit test as implemented in the ALLELE Procedure in SAS.

The repeated measures analyses were carried out using a mixed linear model as implemented in the MIXED Procedure in SAS. A mixed linear model is a generalization of the standard linear model where the data are permitted to exhibit correlation and nonconstant variability, thereby providing the flexibility of modeling the variances and covariances of the data, in addition to the means. The covariance structure for the lung function data was specified using an unstructured model, which provided the best fit for the data after testing other covariance matrices (compound symmetry, spatial exponential, autoregressive, and autoregressive-heterogeneous). All models adjusted for time (in weeks) and contained a SNP × time interaction term. However, since the time interaction terms were not significant in any of the models, they were dropped from the final models presented in the results. Multiple testing for the single SNP association analyses was addressed by controlling the false discovery rate (FDR) using the method of Benjamini and Liu[15,16] (FDR tool available at . Control of the FDR was set at the 0.05 threshold.

Haplotype associations were explored with score tests that account for linkage phase ambiguity [17]. The score tests, derived from generalized linear models, are used for global tests of association, as well as haplotype-specific tests. The haplo.stats program implements the methods of Schaid et al, and was used for these analyses. Haplotypes were imputed and frequencies estimated using the modified EM algorithm estimation facility in haplo.stats. Analyses were run with and without adjustment for nongenetic factors. We modified the method to include data from individuals with partially missing marker information. The minimum haplotype frequency was set at 2.5%. As previously reported [8] in an analysis of 59 SNPs across the genome, we found no evidence for population stratification in either population.

## Results

**Figure 2:**
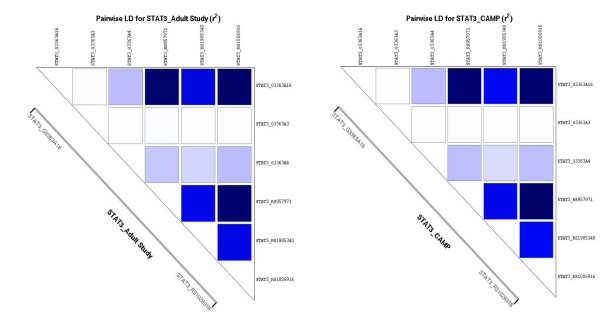
**Linkage disequilibrium (LD) plots among the six *STAT3 *SNPs for both cohorts.**LD is expressed as the r^2 ^statistic.

Baseline characteristics and genotype frequencies of the six SNPs in both cohorts are shown in Table 2. G3363a3 in the Adult Study was the only SNP out of Hardy-Weinberg equilibrium, due to one rare individual who was homozygous for the minor allele. Genotype frequencies were also similar for both cohorts. Figure 2 plots the LD patterns among the six SNPs, with the corresponding r^2 ^values in Table 3. The LD patterns are similar in the two cohorts.

**Table 2:** Baseline characteristics and genotype frequencies for the 6 SNPs in both cohorts.

		**Adult Study N = 401**		**CAMP N = 652**	
Age, mean(sd)		39.2 (13.7)		8.9 (2.1)	
Gender, male, n(%)		171 (42.8)		393 (60.3)	
FEV_1 _(%predicted), mean (sd)		71.7 (12.8)		94.3 (14.2)	
Number of positive skin tests, median (minimum, maximum)		---		4 (0, 18)	
Eosinophil count, geometric mean (sd)		---		5.86 (2.06)	
IgE, geometric mean (sd)		---		404.0 (4.9)	
Paternal asthma, n(%)		---		125 (19.2)	
Maternal asthma, n(%)		---		164 (25.2)	
Maternal cigarette smoking during pregnancy, n(%)		---		88 (13.5)	
SNP genotypes, n(%)			HWE p-value		HWE p-value
G3363a16	GG	145 (36.2)		245 (37.6)	
	GT	153 (38.0)	0.6	291 (44.6)	0.7
	TT	46 (11.5)		80 (12.3)	
	missing	57 (14.2)		36 (5.5)	
G3363a3	GG	342 (85.2)		582 (89.3)	
	GA	1 (0.2)	0.004	2 (0.3)	1.0
	AA	1 (0.2)		0	
	missing	57 (14.2)		68 (10.4)	
G3363a4	GG	137 (34.0)		218 (33.4)	
	GC	175 (43.8)	0.3	291 (44.6)	0.5
	CC	70 (17.5)		85 (13.0)	
	missing	19 (4.8)		58 (8.9)	
rs1026916	GG	164 (41.0)		227 (34.8)	
	GA	181 (45.0)	0.8	233 (35.7)	0.2
	AA	52 (13.0)		74 (11.3)	
	missing	4 (1.0)		118 (18.1)	
rs1905340	CC	203 (50.8)		254 (39.0)	
	CA	159 (39.5)	1.0	191 (29.3)	0.6
	AA	30 (7.5)		41 (6.3)	
	missing			166 (25.5)	
rs957971	CC	156 (39.0)		242 (37.1)	
	CG	172 (42.8)	0.6	260 (39.9)	1.0
	GG	54 (13.5)		70 (10.7)	
	missing			80 (12.3)	

**Table 3:** Linkage disequilibrium (r^2^) among the six *STAT3 *SNPs*.

**Adult Study**	**G3363a16**	**G3363a3**	**G3363a4**	**rs1026916**	**rs1905340**	**rs957971**
**CAMP**						
**G3363a16**		0.0089	0.1662	0.9804	0.6985	0.9936
**G3363a3**	0.0016		0.0003	0.0076	0.0018	0.0017
**G3363a4**	0.1668	0.0012		0.1418	0.1051	0.1666
**rs1026916**	0.9754	0.0037	0.1627		0.6693	0.9718
**rs1905340**	0.6563	0.0002	0.0888	0.6644		0.6910
**rs957971**	0.9734	0.0036	0.1615	0.9854	0.6536	

*r2 between SNPs for the Adult Study are above the diagonal and for the CAMP Study are below the diagonal

**Figure 3:**
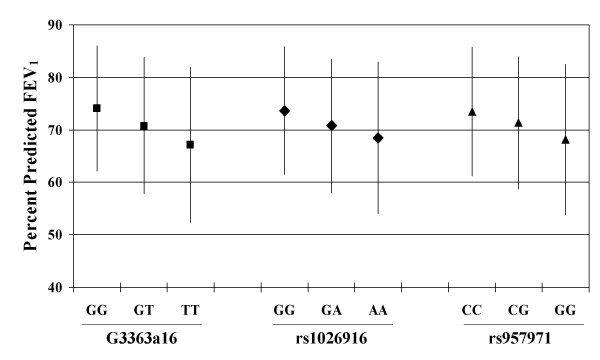
**The association between *STAT3 *SNPs and FEV_1_**. Mean (± sd) percent predicted FEV_1 _in the Adult Study plotted against genotype for each SNP. Additive genetic models were statistically significant for each SNP: p = 0.0007, 0.0043, and 0.007, respectively for G3363a16, rs1026916, and rs957971.

Table 4 and Figure 3 show the results of linear regression models for baseline PPFEV_1_. In the Adult Study, three SNPs showed an effect on lung function. For G3363a16, subjects who were homozygous TT had PPFEV_1 _levels that were 6.85% lower than levels for GG homozygotes. Likewise, for rs1026916 and rs957971, subjects who were homozygous for the minor allele had lower PPFEV_1 _values than did subjects who were homozygous for the major allele. These results remained significant after controlling for the FDR (i.e. the p-values associated with each SNP were smaller than the FDR threshold p-value, thus we reject the null hypothesis of no significant association). In the CAMP trial, only rs1026916 was significantly associated with baseline PPFEV_1_, however, this did not remain statistically significant after controlling for the FDR (i.e. the p-value was greater than the FDR threshold p-value, thus we are unable to reject the null hypothesis of no significant association). Although not statistically significant, the direction of the changes in lung function associated with variation in G3363a16 and rs957971 paralleled those of the Adult Study. Similar results were obtained when we used raw pre-BD FEV_1 _measures, adjusted for age, sex, and height. Additional control for exposure to maternal smoking in utero or post-natal maternal or paternal smoking did not change the results. Interactions between individual SNPs and parental smoking variables were not significant. There was no significant association of any of the SNPs with forced vital capacity.

**Table 4:** Results of linear regression models for baseline PPFEV_1_*

	**Adult Study**			**CAMP**		
**Stat3 SNPs**	**β (se)†**	**Additive genetic model p-value‡**	**FDR threshold (decision§)**	**β (se)†**	**Additive genetic model p-value‡**	**FDR threshold (decision§)**

G3363a16						
Intercept (GG)	74.03 (1.06)			95.67 (0.92)		
GT	-3.35 (1.48)	0.0007	0.007	-1.93 (1.25)	0.1	0.02
TT	-6.85 (2.15)		(reject)	-2.08 (1.84)		(accept)
G3363a4						
Intercept (GG)	70.76 (1.10)			94.19 (0.98)		
GC	1.18 (1.47)	0.3	0.05	1.06 (1.29)	0.6	0.03
CC	2.04 (1.89)		(accept)	-2.04 (1.86)		(accept)
rs1026916						
Intercept (GG)	73.59 (0.93)			96.21 (0.95)		
GA	-2.90 (1.38)	0.004	0.01	-2.21 (1.33)	0.02	0.007
AA	-5.22 (2.03)		(reject)	-4.11 (1.90)		(accept)
rs1905340						
Intercept (CC)	73.04 (0.89)			95.52 (0.89)		
CA	-3.00 (1.34)	0.04	0.03	-1.18 (1.36)	0.8	0.05
AA	-2.77 (2.49)		(accept)	0.70 (2.39)		(accept)
rs957971						
Intercept (CC)	73.48 (1.02)			95.56 (0.01)		
CG	-2.10 (1.41)	0.007	0.02	-2.07 (1.26)	0.08	0.01
GG	-5.34 (2.00)		(reject)	-2.64 (1.91)		(accept)

* Results are from individual linear regression models.

† β 's and p-values are from linear regression models with dummy-coded genotype categories.

‡ p-values refer to the categorical genotype variable (0, 1, 2), for the number of minor alleles present.

§Refers to the decision of whether to accept or reject the null hypothesis of no genotype effect. Please see text for further details.

We then took the SNPs that were significant in the baseline analysis and performed a repeated measures analysis in each cohort, to take advantage of the multiple measures of PPFEV_1_. In the Adult Study, all 401 participants had complete data on PPFEV_1 _at all three time points. In the repeated measures analysis, results were similar to those of the analyses on baseline PPFEV_1 _(Table 5), although only the results for G3363a16 and rs957971 remained significant after control for FDR. Since there were no differences in PPFEV_1 _levels at baseline or at any time point between the two treatment groups, we did not additionally control for treatment group in the multivariable longitudinal models. For CAMP, 6358 observations were available for analysis: 557 (85.3%) children had complete information for the 10 time points, 56 (8.6%) had information for 9 time points, 18 (2.8%) had information for 8 time points, and 22 (3.4%) had information for 3 to 7 time points. In CAMP, rs1026916 was associated with lung function over the 4 years of observation, and stratified analyses showed similar effects of the SNP across treatment groups. Although treatment group had a significant effect on PPFEV_1 _over time, a formal test for interaction between SNP and treatment group was not significant. Thus, treatment group was left as a covariate in the final models. In both cohorts, the SNP × time interaction terms were not significant, meaning that the effects of the individual SNPs did not vary over the time of each study.

**Table 5:** Effects of Stat3 SNPs on repeated measures of PPFEV_1 _over time*

	**Adult Study**			**CAMP**	
**Stat3 SNPs**	**β (se)**	**Additive genetic model p-value‡**	**FDR threshold (decision§)**	**β (se)**	**Additive genetic model p-value‡**

G3363a16					
Intercept (GG)	73.85 (1.21)				
GT	-2.72 (1.69)	0.008	0.009	---	---
TT	-6.33 (2.46)		(reject)		
rs1026916					
Intercept (GG)	73.46 (1.13)			97.01 (0.93)	
GA	-2.25 (1.56)	0.028	0.017	-0.19 (0.99)	0.02
AA	-4.74 (2.30)		(accept)	-4.24 (1.49)	
rs957971					
Intercept (CC)	73.36 (1.16)				
CG	-1.51 (1.60)	0.036	0.038	---	---
GG	-5.04 (2.29)		(reject)		

* β's obtained from mixed models of repeated measurements of percent predicted FEV_1 _over time. All models are adjusted for time in weeks, with a maximum of 3 observations for the Adult Study, and 10 observations for the CAMP study. The model for CAMP is adjusted additionally for treatment group.

§Refers to the decision of whether to accept or reject the null hypothesis of no genotype effect. Please see text for further details.

Haplotype analyses on baseline PPFEV_1 _were performed for each cohort. Table 6 presents the results for the Adult Study. There were five haplotypes that had frequencies above 2.5%. The global statistic was significant at p = 0.02. Haplotype 5, which is comprised of the major alleles for G3363a16, rs957971, and rs1026916, was positively associated with PPFEV_1_, meaning that this haplotype was associated with higher PPFEV_1 _values. On the other hand, haplotype 1, which contained the minor alleles of these three SNPs, was negatively associated with PPFEV_1_. These results were consistent with those of the single SNP analysis. Haplotype analysis in CAMP was also consistent with the single SNP analyses, but did not reach statistical significance (data not shown).

**Table 6:** Association of haplotypes in the *STAT3 *gene and baseline PPFEV_1 _in the Adult Study.

**Haplotypes**							**Frequency**	**Haplotype-Specific Score Statistic**	**p-value**
	G3363a4	G3363a3	**G3363a16**	**rs957971**	rs1905340	**rs1026916**			
(1)	C	G	T	G	A	A	0.04501	-1.91	0.05
(2)	G	G	T	G	A	A	0.23441	-1.73	0.08
(3)	G	G	T	G	C	A	0.06895	-1.60	0.1
(4)	G	G	G	C	C	G	0.27914	0.96	0.3
(5)	C	G	G	C	C	G	0.35557	2.58	0.01

Global Score Statistics:

Global Statistic: 13.3, df = 5

Global p-value = 0.02

## Discussion

This is the first report of an association between SNPs in the *STAT3 *gene and lung function in human populations. In this study, we show an association between SNPs in the *STAT3 *gene and FEV_1 _among asthmatics. These results are reasonably robust and are consistent in a cohort of adult asthmatics and a cohort of childhood asthmatics. Although only one SNP was significant in both cohorts, the direction of the effect of each individual SNPs was similar in the two cohorts. These effects were seen when we analyzed baseline FEV_1 _and the repeated measures of FEV_1 _over 8 weeks in the adult cohort and over 4 years in the childhood asthma cohort. In the cohort of childhood asthmatics, these effects were independent of parental smoking and asthma treatment group.

STAT proteins comprise a family of transcription factors latent in the cytoplasm, that are activated by a series of extracellular signaling proteins such as cytokine, growth factors, and hormones that bind to specific cell-surface receptors. The resulting signal transduction pathways permit them to play different roles in normal physiological cell processes, such as differentiation, proliferation, apoptosis, and angiogenesis [18,19]. Whereas other members of this gene family have generally demonstrated specificity in individual signaling pathways, *STAT3 *is deployed in various, sometimes disparate, physiological processes [6], including cell growth and differentiation [20], apoptosis [21], and anti-inflammatory processes mediated by IL-10 [22], to name a few. Additionally, while the different functions of the members of this gene family have been elucidated via targeted gene ablation, ablation of *STAT3 *leads to embryonic lethality in transgenic mice [23], underscoring its importance in embryogenesis.

In the lung, the function of *STAT3 *has not been fully elucidated. However, *STAT3 *appears to play a role in the regulation of surfactant [24,25], and in the inflammatory response in acute lung injury [26,27]. Additionally, *STAT3 *is an important mediator in the pro-inflammatory effects of the Th2 cytokine IL-13 on lung myofibroblasts [7,28].

It is plausible that our results are due to an effect of the *STAT3 *gene during the embryonic stage of lung development. However, the results in the CAMP cohort show that this effect, if present, is likely a small one. On the other hand, *STAT3 *had stronger effects on lung function in the adult asthmatics, and a potential explanation for our findings is that *STAT3 *interacts with pro-inflammatory environmental stimuli, such as tobacco smoke, to affect FEV_1 _level. It is known that at least three factors determine lung function at a particular point in adult life: (1) the maximally attained level of lung function; (2) the onset of decline of lung function (or alternatively, the duration of the plateau phase); and (3) the rate of decline of lung function [29]. Whether *STAT3 *affects only a particular phase of lung growth or decline, or affects all phases remains to be seen. Furthermore, it needs to be determined whether this effect of *STAT3 *on FEV_1 _level is unique to asthmatics or also applies to non-asthmatics.

Because the effects of *STAT3 *were stronger in the Adult Study, we additionally hypothesized that *STAT3 *may interact with environmental exposures to cause a decrement in lung function. Since cigarette smoking is established as the major environmental risk factor for low lung function[30], we hypothesized that exposure to cigarette smoke (either personal smoking or environmental tobacco smoke) could potentially interact with *STAT3*. We were unable to test the interaction between *STAT3 *and smoking in the Adult Study, because participants were non-smokers. In CAMP, we did not see a significant interaction effect between the individual SNPs and parental smoking variables (in utero smoke exposure, maternal smoking, paternal smoking), and too few children smoked to permit any meaningful interaction analyses. It is also possible that other environmental exposures that we did not measure could be interacting with *STAT3*.

A limitation of our study is the lack of complete sequence information on the gene. The sequencing efforts focused only on the exons, thus limiting our knowledge of the full linkage disequilibrium pattern of the gene. Furthermore, since all the SNPs we successfully genotyped were in intronic regions of the gene, it is likely that these are not responsible for the association but are rather in linkage disequilibrium with the functional variant of this gene, or with variants in another gene. Studying other populations, such as general population samples or occupational cohorts with exposure information, may help elucidate the effects of this gene on FEV_1_. Additionally, more functional studies of SNPs in *STAT3 *are needed to elucidate its role in lung function development.

Airway hyperresponsiveness was not assessed in the Adult Study. However, the subjects had a physician diagnosis of moderate to severe asthma (as evidenced by the levels of lung function), were on inhaled steroids at baseline, and had a significant bronchodilator response to albuterol. The combination of a physician diagnosis and bronchodilator response is a reasonable definition of asthma in genetic studies[31]. Furthermore, significant smoking and non-asthma respiratory disorders were excluded. Additionally, we did not have information on allergy outcomes in the Adult Study, either. In CAMP, we performed additional analyses, however, there were no associations between any of the *STAT3 *SNPs or either serum IgE level or skin test reactivity.

We controlled for multiple testing by controlling the false discovery rate. An additional strategy we took to minimize the effect of multiple testing is by performing a screening analysis in the Adult Study, then performing a replicate analysis in CAMP. Population stratification is another potential concern [32]; thus, we included only Caucasian subjects in this analysis. Furthermore, in previous testing utilizing a panel of 59 random markers, we found no evidence for stratification in the Caucasian subjects in either of these two cohorts [8].

## Conclusion

We have shown that polymorphisms in *STAT3 *are associated with FEV_1 _in asthmatics. We show these effects both in a cohort of adult asthmatics and in a cohort of childhood asthmatics. In both cohorts, we excluded gross population stratification by testing with a panel of random markers. The precise mechanism for the effects of this gene on FEV_1 _remains unknown. However, while we see an association between SNPs in this gene and FEV_1 _in young asthmatics, the effects were stronger in the adult asthmatics, suggesting a role of *STAT3 *in chronic inflammatory pathways that may have an impact on lung growth and decline.

## Authors' contributions

AAL participated in the conceptualization of the analysis, designed the analysis, performed the analyses and interpretation of results, and drafted the manuscript. KGT participated in the selection and genotyping of SNPs, conception and design of the study, acquisition of the data, and drafting and critically revising the manuscript. SL participated in the statistical analysis of the data and in critically revising the manuscript. RL participated in the conception and design of the analysis, and in critically revising the manuscript. BGR participated in the preparation of the data for analysis and in critically revising the manuscript. SG participated in the selection and genotyping of SNPs and in critically revising the manuscript. ESS participated in the design of the analysis and in critically revising the manuscript. STW conceived of the study, participated in its design and its coordination, participated in acquisition of the data, and participated in critically revising the manuscript.

## Grant Support

This work was supported by: U01 HL065899 – *The Pharmacogenetics of Asthma Treatment*; the *Childhood Asthma Management Program (CAMP) *by contracts N01-HR-16044, 16045, 16046, 16047, 16048, 16049, 16050, 16051, and 16052; and the *CAMP Genetics Ancillary Study *by P01 HL067664, all awarded by the NHLBI.
